# Physical fitness and plasma leptin in women with recent gestational diabetes

**DOI:** 10.1371/journal.pone.0179128

**Published:** 2017-06-13

**Authors:** C. Gar, M. Rottenkolber, H. Grallert, F. Banning, I. Freibothe, V. Sacco, C. Wichmann, S. Reif, A. Potzel, V. Dauber, C. Schendell, N. N. Sommer, B. Wolfarth, J. Seissler, A. Lechner, U. Ferrari

**Affiliations:** 1 Diabetes Research Group, Medizinische Klinik IV, Klinikum der Universitaet Muenchen, Munich, Germany; 2 CCG Type 2 Diabetes, Helmholtz Zentrum München, Munich, Germany; 3 Deutsches Zentrum für Diabetesforschung (DZD), Neuherberg, Germany; 4 Institut für klinische Radiologie, Klinikum der Universitaet Muenchen, Munich, Germany; 5 Humboldt Universitaet/Charité, Universitaetsmedizin Berlin, Abteilung Sportmedizin, Berlin, Germany; Shanghai Diabetes Institute, CHINA

## Abstract

**Aims/Hypothesis:**

Low physical fitness (PF) is a risk factor for type 2 diabetes mellitus (T2D). Women with a history of gestational diabetes (GDM) are at risk for T2D at a young age, but the role of PF in this population is not clear. PF has also been found to correlate inversely with plasma leptin in previous studies. Here, we examine whether women who had GDM have lower PF than women after a normoglycemic pregnancy and, second, whether PF is associated with plasma leptin, independently of body fat mass.

**Methods:**

Cross-sectional analysis of 236 participants in the PPSDiab Study (cohort study of women 3–16 months after delivery, 152 after gestational diabetes (pGDM), 84 after normoglycemic pregnancy (control subjects); consecutively recruited 2011–16); medical history, physical examination with bioelectrical impedance analysis (BIA), whole body magnetic resonance imaging (MRI) (n = 154), 5-point oral glucose tolerance test, cardiopulmonary exercise testing, clinical chemistry including fasting plasma leptin; statistical analysis with Mann–Whitney U and t -test, Spearman correlation coefficient, multiple linear regression.

**Results:**

Women pGDM had lower maximally achieved oxygen uptake (VO_2peak_/kg: 25.7(21.3–29.9) vs. 30.0(26.6–34.1)ml/min/kg; total VO_2peak_: 1733(1552–2005) vs. 1970(1767–2238)ml/min; p<0.0001 for both), and maximum workload (122.5(105.5–136.5) vs. 141.0(128.5–159.5)W; p<0.0001). Fasting plasma leptin correlated inversely with PF (VO_2peak_/kg ρ = -0.72 p<0.0001; VO_2peak_ ρ = -0.16 p = 0.015; max. load ρ = -0.35 p<0.0001). These associations remained significant with adjustment for body mass index, or for body fat mass (BIA and MRI).

**Conclusions/Interpretation:**

Women with a recent history of GDM were less fit than control subjects. Low PF may therefore contribute to the risk for T2D after GDM. This should be tested in intervention studies. Low PF also associated with increased leptin levels–independently of body fat. PF may therefore influence leptin levels and signaling. This hypothesis requires further investigation.

## Introduction

Gestational diabetes (GDM) is a transient disturbance of glucose metabolism, with a prevalence ranging from 1.1% to 24.3%, depending on diagnostic criteria [1]. It is a strong risk marker for subsequent type 2 diabetes mellitus (T2D) [2, 3], and women with recent GDM already show many metabolic characteristics associated with T2D [4].

Physical fitness (PF) and activity are major determinants of diabetes risk [5]. Muscle glucose uptake is particularly important for postprandial glucose tolerance, and regular exercise increases the insulin sensitivity of skeletal muscle [6]. Additionally, glucose metabolism of trained individuals with high PF is also probably affected more indirectly by altered interorgan communication [7, 8]. The gold-standard methodology to quantify PF is cardiopulmonary exercise testing. To our knowledge, this has not been done in women with recent GDM.

The hormone leptin is produced by the adipose tissue as an indicator of the amount of stored energy (as fat) [9]. Its plasma level correlates closely with the quantity of fat tissue and therefore also the body mass index (BMI) [10]. Leptin mainly acts centrally, predominantly to control appetite [11]. It is also required for an adequate neuroendocrine function, i.e. secretion of sexual hormones, growth and thyroid hormone and has been shown to influence glucose metabolism by increasing insulin sensitivity [9, 11–14]. This mainly occurs through an activation of the sympathetic nervous system and by direct action on peripheral leptin receptors in skeletal muscle [13]. Surprisingly, however, high plasma leptin levels often coexist with insulin resistance in human subjects. We and other groups have even found a negative correlation between fasting plasma leptin and insulin sensitivity after adjustment for BMI [4, 5]. Given the insulin-sensitizing effect of leptin, this finding requires dysfunction or saturation of leptin signaling, phenomena often summarized under the term “leptin resistance”.

An additional factor that may influence leptin sensitivity is physical fitness. Low PF has been found to be associated with high leptin levels in previous studies [5, 15–17] and exercise interventions can lead to a reduction in plasma leptin [18].

Based on these lines of evidence, we examined two research questions in an observational study of young women. First, whether women with a recent history of GDM have lower PF than appropriate control subjects. Second, whether PF is associated with the plasma leptin level after adjustment for BMI or body fat mass. Positive answers to both questions would provide initial evidence that increasing PF in women with recent GDM by an exercise intervention may have the double benefit of reducing both insulin and leptin resistance.

## Material and methods

### Study design and participants

Women included in the present cross-sectional analysis were participants of the prospective, mono-center observational study PPSDiab (“Prediction, Prevention and Subclassification of type 2 diabetes”) enrolled between November 2011 and May 2016. The study population consists of women with GDM during their last pregnancy (pGDM) and women following a normoglycemic pregnancy (controls) in the ratio 2:1. The cohorts were recruited consecutively from the Diabetes Center and the obstetrics department of the University Hospital (Klinikum der Universität München) in Munich, Germany.

Eligible women were premenopausal and within 3 to 16 months after a singleton or twin (n = 9) pregnancy with live birth(s). The diagnosis of GDM was based on a 75g oral glucose tolerance test (OGTT) after the 23rd week of gestation. The cut-off values for GDM were 92/180/153 mg/dl plasma glucose following the International Association of the Diabetes and Pregnancy Study (IADPSG) recommendations [19]. Women were eligible to participate as controls if they had no history of GDM in any previous pregnancy and either a normal 75g OGTT or a normal 50g screening OGTT (<135 mg/dl plasma glucose after 1 hour, n = 10) after the 23rd week of gestation. We included controls with only a screening OGTT because in Germany a 2-Step approach of testing for GDM is widely used and women without known risk-factors for GDM may only receive the 50g test.

Exclusion criteria for this study were alcohol or substance abuse, pre-pregnancy diabetes, and chronic diseases requiring systemic medication (except for hypothyroidism (n = 52), mild hypertension (n = 4), gastroesophageal reflux (n = 2), and history of pulmonary embolism resulting in Rivaroxaban prophylaxis (n = 1)).

Written informed consent was obtained from all study participants and the protocol was approved by the ethical review committee of the Ludwig-Maximilians-Universität.

All data used in this analysis were collected at the baseline visit of the PPSDiab study, 3 to 16 months after the index pregnancy.

### Study procedures

After an overnight fast, the women underwent a 5-point 75-g oral glucose tolerance test with measurement of plasma glucose (Glucose HK Gen.3, Roche Diagnostics, Mannheim, Germany), serum insulin (CLIA, DiaSorin LIASON systems, Saluggia, Italy), plasma leptin (ELISA "Dual Range", Merck Millipore, Darmstadt, Germany), high sensitivity c-reactive protein (hs-CRP; wide-range CRP, Siemens Healthcare Diagnostics, Erlangen, Germany) and blood lipids (LDL and HDL cholesterol, triglycerides; enzymatic caloric test, Roche Diagnostics, Mannheim, Germany). Insulin sensitivity index (ISI) was calculated according to Matsuda and DeFronzo (ISI = 10,000/square root of [fasting glucose x fasting insulin] x [mean glucose x mean insulin during OGTT]) [20]. Anthropometric data included body mass, body fat mass (determined by bioelectrical impedance analysis (Tanita BC-418; Tanita Corporation) [21, 22]), height, waist and hip circumference. A detailed description of the study design, anthropometric and clinical measurements as well as methodologies of blood sampling and analysis can be found elsewhere [4].

For determination of PF, cardiopulmonary exercise testing was performed on a bicycle ergometer using the cardiopulmonary exercise testing system MasterScreen CPX (Care Fusion, Höchberg, Germany). Prior to this test, cardiopulmonary health was ascertained from the medical history and clinical examination including auscultation and measurement of resting blood pressure. Due to the heterogeneity of the study cohort regarding physical fitness levels, we used a standardized stepwise ramp protocol for all participants. It consisted of stepwise increments of 25 W every 3 minutes, starting with a reference phase without load. In order to reach a plateau phase of the oxygen curve (levelling-off effect), which is required for determining the maximal possible oxygen uptake of the cardiopulmonary system (VO_2max_), an individualized, steep ramp protocol and a reasonable baseline fitness of the study participant, who also has to be familiar with the test procedure, would have been needed. This was not possible in our study and we therefore determined the peak oxygen uptake before termination of workload (VO_2peak_), a close approximation of VO_2max_ [23]. 12-channel ECG, oxygen uptake, and carbon dioxide exhalation were monitored continuously, while at the end of each increment, capillary lactate was measured using a SuperGL Analyser (Hitado, Möhnesee, Germany), and participants were asked to rate their perceived exertion by pointing to a BORG scale [24]. The test was terminated when the participant was exhausted. A maximal respiratory exchange ratio (RER) of at least 1.05 was required for a valid exercise test.

Study participants were invited to undergo a whole-body magnetic resonance imaging (MRI) measurement (3 Tesla system, Ingenia or Achieva; Philips Healthcare) with determination of total adipose tissue volumes. Three days before MRI study, participants were advised to refrain from heavy exercise. The MRI study protocol has been described previously [4].

### Statistical analysis

All metric and normally distributed variables are reported as mean ± standard deviation; non-normally distributed variables are presented as median (first quartile–third quartile). For group comparisons, the t-test was used for normally and the Mann–Whitney U-test was used for non-normally distributed metric variables. P-values <0.05 were considered to be statistically significant. Spearman correlation coefficient (ρ) was calculated for correlation analysis. Linear regression models (raw and with adjustment for BMI or body fat mass (BIA and MRI), age, months post-delivery) were conducted with the dependent variables (all logarithmized) peak oxygen uptake (“VO_2peak_”), peak oxygen uptake per body weight (“VO_2peak_/kg”) and maximum workload in cardiopulmonary exercise testing (“Max. load”) and “pGDM/control-status” as independent variable. We also calculated raw and adjusted (BMI or body fat mass (BIA and MRI), age, months post-delivery, pGDM/control status) linear regression models with “leptin” (logarithmized) as dependent and “VO_2peak_”, “VO_2peak_/kg” and “Max. load” as independent variables. All statistical calculations were performed using the SAS statistical software package, version 9.3 (SAS Institute Inc., Cary, NC, USA) or R version 3.0.2 (http://www.R-project.org).

## Results

**Fig 1:**
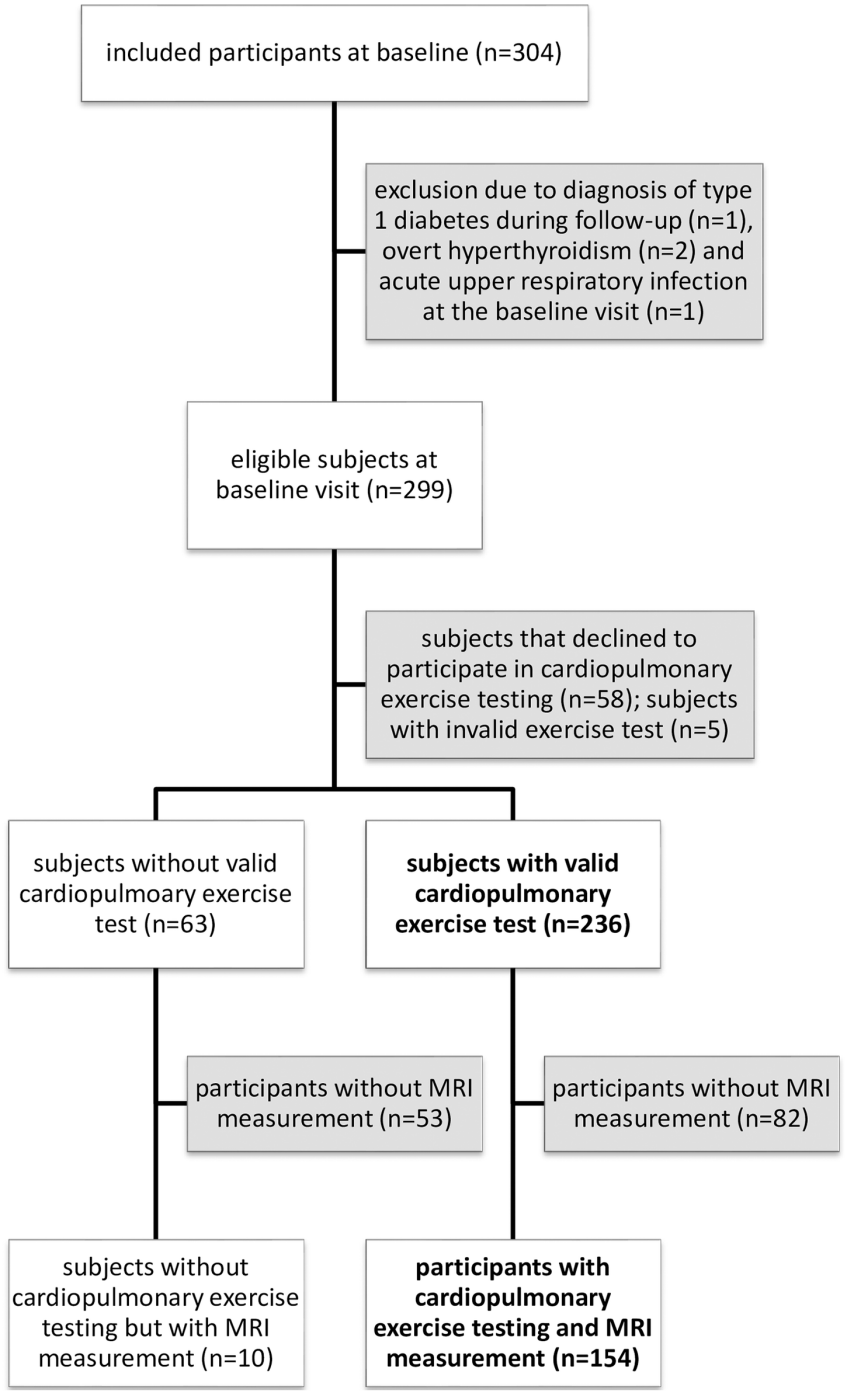
Recruitment flow chart. Cohorts analyzed in this manuscript are shown in bold type. MRI: magnetic resonance imaging.

From November 2011 to May 2016, 304 women were recruited into the PPSDiab study cohort. This analysis focuses on the baseline visit, which was 3 to 16 months after delivery (Fig 1). We excluded five women from this analysis, two because of type 1 diabetes diagnosed during follow-up, two because of overt hyperthyroidism and one because of an acute upper respiratory infection at baseline (Fig 1). 58 women declined to participate in cardiopulmonary exercise testing and 5 were excluded from the analysis due to an invalid exercise test (technical failure in measurement of O_2_/CO_2_ curves: n = 3; failure in measurement of O_2_/CO_2_ curves due to leaky mask: n = 1; unmet exhaustion criteria (low RER): n = 1). Consequently, the final sample consisted of 236 women, 152 women pGDM and 84 control subjects (Fig 1). Women with a valid exercise test were slightly older and less overweight than those without (S1 Table). The proportion of participants with a valid test was comparable in women pGDM and controls.

The baseline characteristics of the final study sample are shown in Table 1. Women pGDM had larger waist circumference, higher values for BMI, fat mass measured in BIA and MRI, blood pressure, hs-CRP, HDL cholesterol, triglycerides, as well as plasma leptin compared with the control group. Fasting and 2-hour plasma glucose were higher and insulin sensitivity index (ISI) was lower in the pGDM group.

**Table 1:** Characteristics of the study sample. BIA: bioelectrical impedance analysis; BMI: body mass index; ISI: insulin sensitivity index; Max. load: maximum workload in cardiopulmonary exercise testing; MRI: magnetic resonance imaging; VO_2peak_: peak oxygen uptake; VO_2peak_/kg: peak oxygen uptake per body mass.

		Total	pGDM	Control subjects	p-value
**Clinical parameter**	**N**	236	152	84	
	**Age [years]**	35.9±4.1	36.2±4.1	35.4±3.9	0.1438
	**Waist circumference [cm]**	80.6±11.3	82.4±12.1	77.4±9.0	0.0004
	**BMI [kg/m**^**2**^**] (missing n = 2)**	25.0±5.4	25.9±5.9	23.4±3.9	0.0001
	**Fat mass in BIA (missing n = 2)**	23.1±10.5	24.6±11.5	20.2±7.8	0.0007
	**Total fat mass in MRI (n = 154)**	25.5±10.8	27.1±11.7	22.6±8.3	0.0059
	**Systolic blood pressure [mmHg]**	117.2±11.4	118.8±11.3	114.1±11.0	0.0020
	**Diastolic blood pressure [mmHg]**	73.4±9.1	74.6±8.9	71.3±9.1	0.0081
	**Months post-delivery**	9.3±2.8	9.3±2.9	9.2±2.6	0.8764
**Laboratory parameter**	**Leptin [ng/ml]**	10.1 (4.9–15.7)	11.5 (6.7–18.8)	6.4 (3.6–11.6)	<0.0001
	**Adiponectin [ng/ml]**	11.6 (8.0–14.9)	10.7 (7.7–15.2)	11.7 (9.1–14.8)	0.3437
	**hs-CRP [mg/dl]**	0.1 (0.0–0.1)	0.1 (0.0–0.3)	0.0 (0.0–0.1)	0.0044
	**LDL cholesterol [mg/dl]**	104.0 (86.0–120.0)	104.0 (86.5–120.0)	104.5 (84.5–118.0)	0.7842
	**HDL cholesterol [mg/dl]**	62.0 (55.0–73.0)	61.0 (52.0–71.0)	64.0 (57.0–73.5)	0.0368
	**Triglycerides [mg/dl]**	67.0 (53.0–89.5)	71.5 (54.5–97.5)	60.0 (50.0–77.5)	0.0041
**Glucose parameter**	**Fasting plasma glucose [mg/dl]**	91.0 (87.0–97.0)	94.0 (89.0–99.0)	89.5 (84.0–92.5)	<0.0001
	**Plasma glucose 2h [mg/dl]**	109.0 (90.0–125.5)	117.5 (101.0–133.5)	91.0 (80.0–108.0)	<0.0001
	**ISI**	5.4 (3.6–7.6)	4.6 (3.0–6.7)	6.9 (5.2–8.7)	<0.0001
**Cardiopulmonary exercise testing parameter**	**VO**_**2peak**_**/kg [ml/min/kg]**	27.6 (22.6–31.3)	25.7 (21.3–29.9)	30.0 (26.6–34.1)	<0.0001
	**VO**_**2peak**_ **[ml/min]**	1828 (1608–2092)	1733 (1552–2005)	1970 (1767–2238)	<0.0001
	**Max. load [W]**	129.0 (110.0–149.5)	122.5 (105.5–136.5)	141.0 (128.5–159.5)	<0.0001

With respect to our first research question (differences in PF between pGDM and control subjects), women pGDM had lower VO_2peak_/kg body weight, total VO_2peak_, and maximum workload compared with the control group (VO_2peak_/kg: 25.7 (21.3–29.9) vs. 30.0 (26.6–34.1), p<0.0001; VO_2peak_: 1733 (1552–2005) vs. 1970 (1767–2238), p<0.0001; max. load: 122.5 (105.5–136.5) vs. 141.0 (128.5–159.5), p<0.0001; Table 1). The associations of group status with VO_2peak_/kg, total VO_2peak_ and maximum workload remained significant after adjustment for BMI. This association was independent of BMI, age and months since delivery as shown by linear regression analyses (Table 2). Substituting body fat mass determined by BIA or MRI for BMI in these models gave comparable results (S2 and S3 Tables).

**Table 2:** Linear regression analysis - dependent variable VO_2peak_/kg (ml/min/kg), VO_2peak_ (ml/min) or Max. load (all logarithmized), independent variable pGDM/control status. BMI: body mass index; CI: confidence interval; Max. load: maximum workload in cardiopulmonary exercise testing; VO_2peak_: peak oxygen uptake; VO_2peak_/kg: peak oxygen uptake per body mass.

VO_2peak_/kg (ml/min/kg)			VO_2peak_			Max. load		
Regression coefficient (95% CI)	p-value	Adjusted R^2^	Regression coefficient (95% CI)	p-value	Adjusted R^2^	Regression coefficient (95% CI)	p-value	Adjusted R^2^
**No adjustment**								
0.18 (0.11–0.24)	<0.0001	0.11	0.12 (0.07–0.17)	<0.0001	0.08	0.16 (0.11–0.21)	<0.0001	0.13
**Adjustment for BMI**								
0.10 (0.05–0.15)	<0.0001	0.50	0.13 (0.07–0.18)	<0.0001	0.08	0.14 (0.09–0.20)	<0.0001	0.15
**Adjustment for BMI, age, and months post-delivery**								
0.10 (0.05–0.15)	<0.0001	0.51	0.13 (0.08–0.18)	<0.0001	0.09	0.15 (0.10–0.20)	<0.0001	0.16

**Fig 2:**
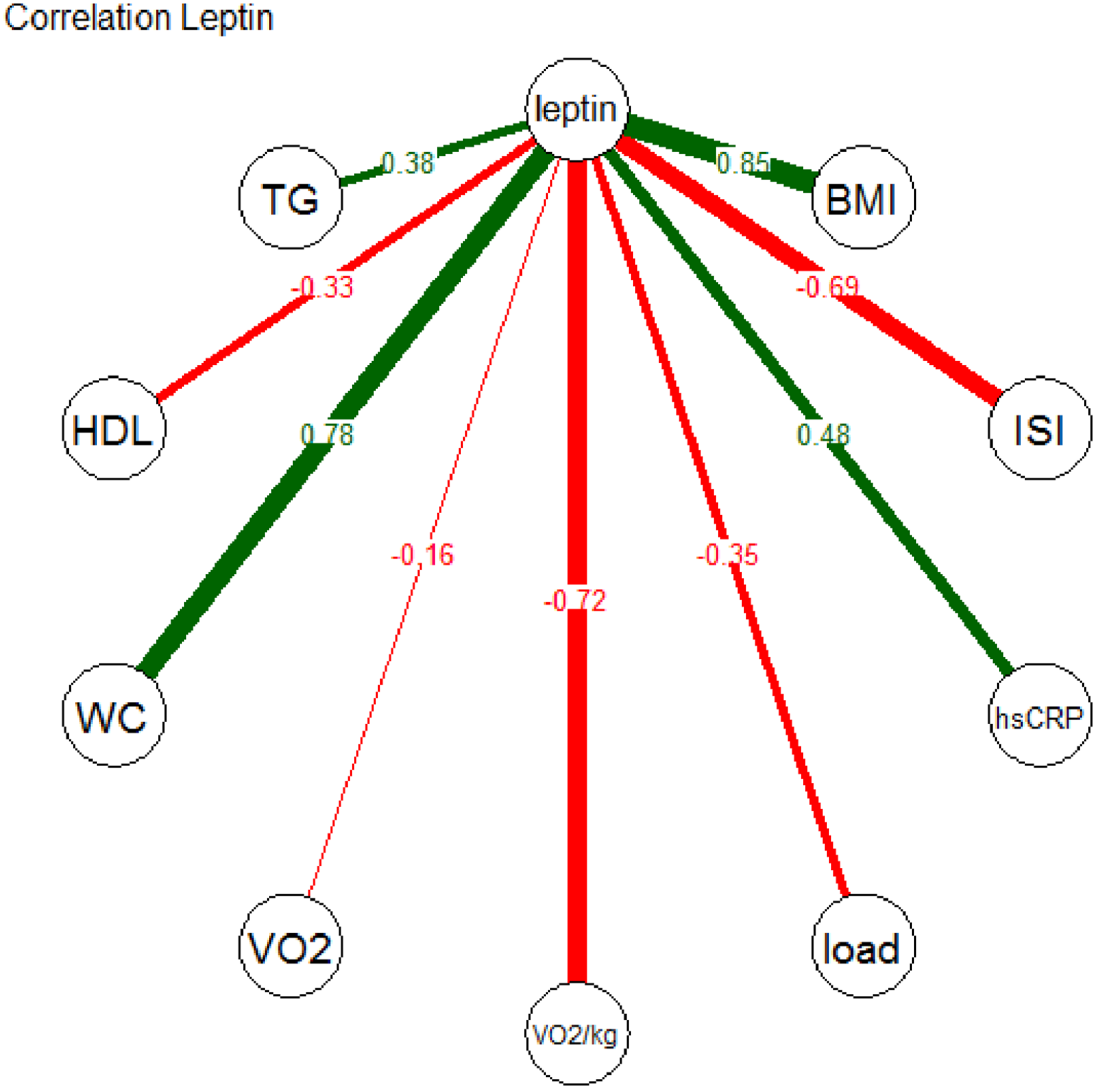
Spearman correlation coefficients for leptin and selected other variables. BMI: body mass index; TG: triglycerides; HDL: HDL cholesterol (mg/dl); ISI: insulin sensitivity index; load: maximum workload in cardiopulmonary exercise testing; VO2: peak oxygen uptake; VO2/kg: peak oxygen uptake per whole body mass; WC: waist circumference (cm); p-value <0.0001 for all, except for leptin with VO2 (p = 0.015).

Concerning the second research question (association of PF and plasma leptin), we found negative correlations between plasma leptin and VO_2peak_/kg, VO_2peak_, and maximum load (Fig 2, VO_2peak_/kg: ρ = –0.72, p<0.0001; VO_2peak_: -0.16, p = 0.015; max. load: ρ = –0.35, p<0.0001).

In order to specifically examine the association of plasma leptin with PF, we calculated multiple linear regression models with adjustment for BMI, or for BMI, pGDM/control status, age, and months post-delivery (Table 3). These analyses confirmed negative associations between plasma leptin and VO_2peak_/kg, VO_2peak_, and maximum workload, which were independent of BMI and the other covariables. Similar models with body fat mass (measured with BIA and in MRI) instead of BMI gave comparable results (S4 and S5 Tables).

**Table 3:** Linear regression analysis - dependent variable leptin (logarithmized). BMI: body mass index; CI: confidence interval; pGDM: previous gestational diabetes; Max. load: maximum workload in cardiopulmonary exercise testing; VO_2peak_: peak oxygen uptake; VO_2peak_/kg: peak oxygen uptake per body mass.

VO_2peak_/kg KG (ml/min/kg KG)			VO_2peak_			Max. load		
Regression coefficient (95% CI)	p-value	Adjusted R^2^	Regression coefficient (95% CI)	p-value	Adjusted R^2^	Regression coefficient (95% CI)	p-value	Adjusted R^2^
**No adjustment**								
-0.09 (-0.10/-0.08)	<0.0001	0.52	-0.001 (-0.001/-0.0002)	0.0007	0.04	-0.01 (-0.02/-0.01)	<0.0001	0.15
**Adjustment for BMI**								
-0.05 (-0.07/-0.04)	<0.0001	0.64	-0.0005 (-0.0007/-0.0003)	<0.0001	0.58	-0.01 (-0.01/-0.01)	<0.0001	0.60
**Adjustment for BMI, pGDM/control status, age, and months post-delivery**								
-0.05 (-0.07/-0.04)	<0.0001	0.64	-0.0004 (-0.0007/-0.0002)	<0.0001	0.58	-0.01 (-0.01/-0.004)	<0.0001	0.59

## Discussion

We measured PF (VO_2peak_/kg, VO_2peak_, and maximum workload during cardiopulmonary exercise testing) in women after gestational diabetes and in women who had a normoglycemic pregnancy. The women after gestational diabetes were less fit, independent of adiposity. We also examined the association of PF and plasma leptin in the whole study cohort. PF was negatively associated with fasting plasma leptin, also after adjustment for BMI or body fat mass.

Women pGDM carry an about 20% risk of developing T2D within 10 years of the index pregnancy [2], and our data suggest that low PF may be one modifiable risk factor contributing to this situation—although our cross-sectional analysis cannot show this directly. Our finding is in line with results from other types of at risk cohorts, e.g., older subjects with impaired glucose tolerance [25]. To our knowledge, PF has not been examined previously by objective measures, such as cardiopulmonary exercise testing, in women with recent GDM. The mean maximum oxygen uptake in women pGDM was over 200 ml/min lower than in control subjects. This represents a clinically meaningful difference. Exercise intervention programs can increase physical fitness [18] and may therefore also be valuable for women pGDM. This should be tested in a study setting.

Further differences between the pGDM and the control group in our study were a higher BMI and worse lipid profiles in the pGDM group. These findings are not surprising and indicate a higher prevalence of the metabolic syndrome in pGDM subjects.

We also saw an inverse association between plasma leptin and PF. Body fat mass remains the main determinant of fasting plasma leptin, but our finding was consistent for three measures of PF, as well as after adjustment for covariates.

Our results regarding an association of leptin with physical fitness are in agreement with work by Cicchella et al. [15], Chu et al. [26] and Miyatake et al. [17]. Chu et al. [26] studied a cohort of 268 male health professionals (age: 47–83 years; mean BMI: normal weight 23.2 kg/m^2^; overweight 27.7 kg/m^2^) but only relied on a questionnaire to estimate physical activity. Cicchella et al. [15] measured VO_2peak_, as we did in this work, but the study cohort consisted of 10- to 12-year old boys. Miyatake et al. [17] found a BMI-independent negative association between leptin and PF in men and between leptin and physical activity in women in a middle aged, healthy Japanese cohort. Only in a 1996 study by Ostlund et al. [10] was the reverse association of leptin and VO_2peak_ lost after adjustment for percent body fat. However, only individuals between 60 and 70 years of age were included in that analysis, which suggests that these results are not representative for the general population. Additionally, correcting for percent body fat may underestimate the role of PF, because of the positive association of PF and muscle mass (with the same fat mass, lower muscle mass leads to a higher percentage of body fat. Adjustment for percent body fat will then result in an over-adjustment of plasma leptin in those with lower muscle mass.). Taken together, an inverse and fat mass-independent correlation of plasma leptin and PF is supported by several studies from the literature and also our own data. Additionally, exercise interventions that increase physical fitness have been shown to also reduce plasma leptin [18].

Poorly trained muscle has a reduced insulin-mediated glucose uptake [27], but hormonal signaling also links PF to insulin sensitivity and glucose homeostasis. This involves myokines but also other hormones like epinephrine, glucocorticoids and, potentially, leptin [6, 28, 29]. An interesting hypothesis, which would be in agreement with our findings, is that PF affects “leptin resistance” where high plasma leptin levels coexist with late satiety and insulin resistance [9, 11, 30]. Several mechanisms have been implicated in this phenomenon [9, 11, 12], most prominently the saturation of the leptin transport system across the blood–brain barrier (BBB) and impaired intracellular signaling downstream of the leptin receptor [11, 31]. One possibility that could link PF to a reduction in “leptin resistance” is the finding that the transport of leptin across the BBB is increased by epinephrine [32–34]. Achieving and maintaining fitness requires regular exercise, which acutely increases plasma epinephrine with each workout [35]. As a consequence, leptin transport across the BBB and its central effects would be enhanced [34].

However, several alternative explanations can be found for the observed reverse association between PF and plasma leptin, e.g., a direct muscle–adipose tissue interaction or an influence of leptin resistance on central rewarding systems that promote voluntary physical activity [36]. Further studies of the effects of changes in PF and of acute and chronic exercise on plasma leptin levels, leptin transport across the BBB and central responses to leptin will be necessary to clarify this issue.

Strengths of this study include its homogeneous, all-female cohort with a small age range and very little concomitant disease and medication. Additionally, PF was measured by the gold standard method of cardiopulmonary exercise testing.

The homogeneous cohort in this study is also one of its weaknesses, as it precludes the generalization of our findings to other populations. The cross-sectional observational design of this analysis does not permit the investigation of cause–effect relationships. In our cohort, women with a valid exercise test were leaner than those who declined to participate in or did not complete exercise testing. This was true for both study groups and therefore probably did not bias our results. The cohort with a valid test also still covered a BMI-range from 18 to 44 kg/m^2^.

In conclusion, our findings suggest that poor PF may contribute to the T2D risk of women with recent GDM. Additionally, our results support the hypothesis of a link between PF and leptin signaling. Specific studies on this issue in humans and animal models are certainly needed to confirm this assumption and, if true, elucidate the relevant pathways. Such studies seem warranted because leptin resistance is probably involved in the pathophysiology of obesity as well as of impaired glucose metabolism [37].

## Supporting information

S1 Table Characteristics of the study sample. BIA: bioelectrical impedance analysis; BMI: body mass index; ISI: insulin sensitivity index; MRI: magnetic resonance imaging.
(TIF)

S2 Table Linear regression analysis - dependent variable VO_2peak_/kg (ml/min/kg), VO_2peak_ or Max. load (all logarithmized), independent variable pGDM/control status. BIA: bioelectrical impedance analysis; CI: confidence interval; Max. load: maximum workload in cardiopulmonary exercise testing; VO_2peak_: peak oxygen uptake; VO_2peak_/kg: peak oxygen uptake per body mass.
(TIF)

S3 Table Linear regression analysis - dependent variable VO_2peak_/kg (ml/min/kg), VO_2peak_ or Max. load (all logarithmized), independent variable pGDM/control status. Analysis of participants with MRI data (n = 154). CI: confidence interval; Max. load: maximum workload in cardiopulmonary exercise testing; MRI: magnetic resonance imaging; VO_2peak_: peak oxygen uptake; VO_2peak_/kg: peak oxygen uptake per body mass.
(TIF)

S4 Table Linear regression analysis - dependent variable leptin (logarithmized), independent variable VO_2peak_/kg (ml/min/kg), VO_2peak_ or Max. load. BIA: bioelectrical impedance analysis; CI: confidence interval; pGDM: previous gestational diabetes; Max. load: maximum workload in cardiopulmonary exercise testing; VO_2peak_: peak oxygen uptake; VO_2peak_/kg: peak oxygen uptake per body mass.
(TIF)

S5 Table Linear regression analysis - dependent variable leptin (logarithmized), independent variable VO_2peak_/kg (ml/min/kg), VO_2peak_ (logarithmized) or Max. load (logarithmized). Analysis of participants with MRI data (n = 154). CI: confidence interval; Max. load: maximum workload in cardiopulmonary exercise testing; MRI: magnetic resonance imaging; pGDM: previous gestational diabetes; VO_2peak_: peak oxygen uptake; VO_2peak_/kg: peak oxygen uptake per body mass.
(TIF)

## Abbreviations

BBB: blood–brain barrier
GDM: gestational diabetes
hs-CRP: high sensitivity c-reactive protein
ISI: insulin sensitivity index
PF: physical fitness
pGDM: previous gestational diabetes
RER: respiratory exchange ratio
T2D: type 2 diabetes mellitus
TG: triglycerides
VO_2peak_: peak volume of oxygen uptake
