# Supplementary figures and images for: Physical fitness and plasma leptin in women with recent gestational diabetes
Source: PLoS One. 2017 Jun 13;12(6):e0179128. doi: 10.1371/journal.pone.0179128 (PMC5469459; doi:10.1371/journal.pone.0179128)

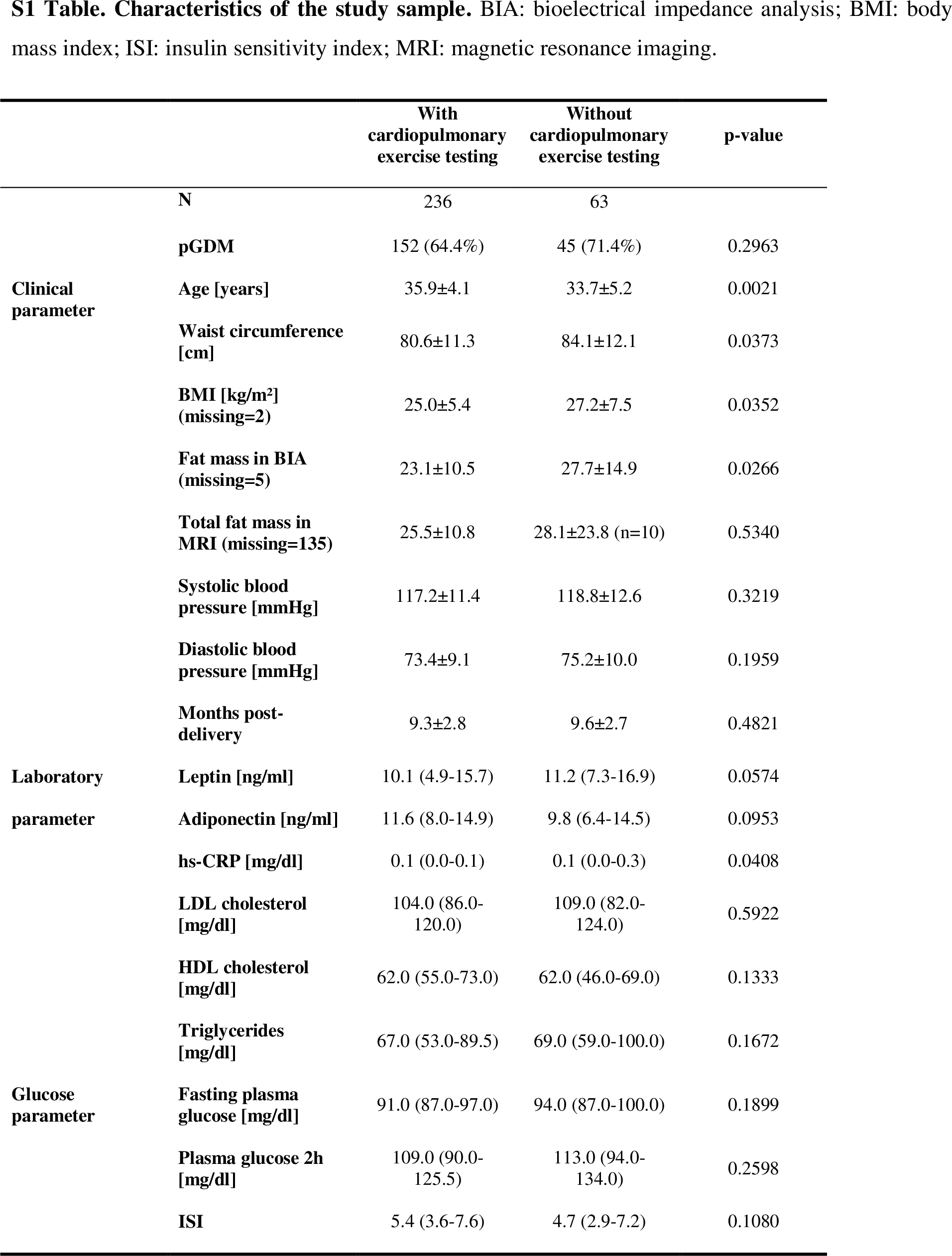

Supplement: S1 Table — BIA: bioelectrical impedance analysis; BMI: body mass index; ISI: insulin sensitivity index; MRI: magnetic resonance imaging. (TIF) [file pone.0179128.s001.tif]

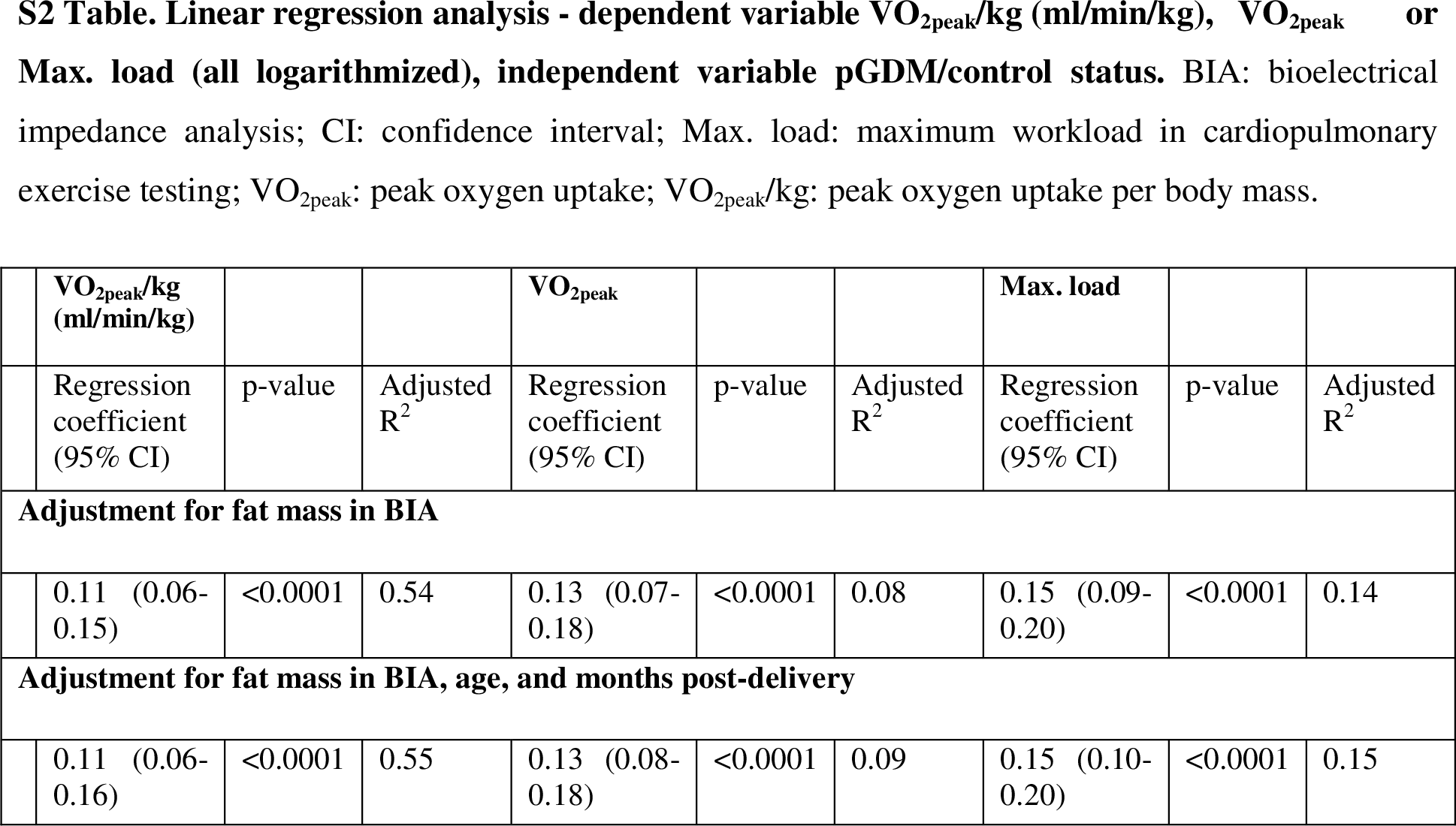

Supplement: S2 Table — BIA: bioelectrical impedance analysis; CI: confidence interval; Max. load: maximum workload in cardiopulmonary exercise testing; VO2peak: peak oxygen uptake; VO2peak/kg: peak oxygen uptake per body mass. (TIF) [file pone.0179128.s002.tif]

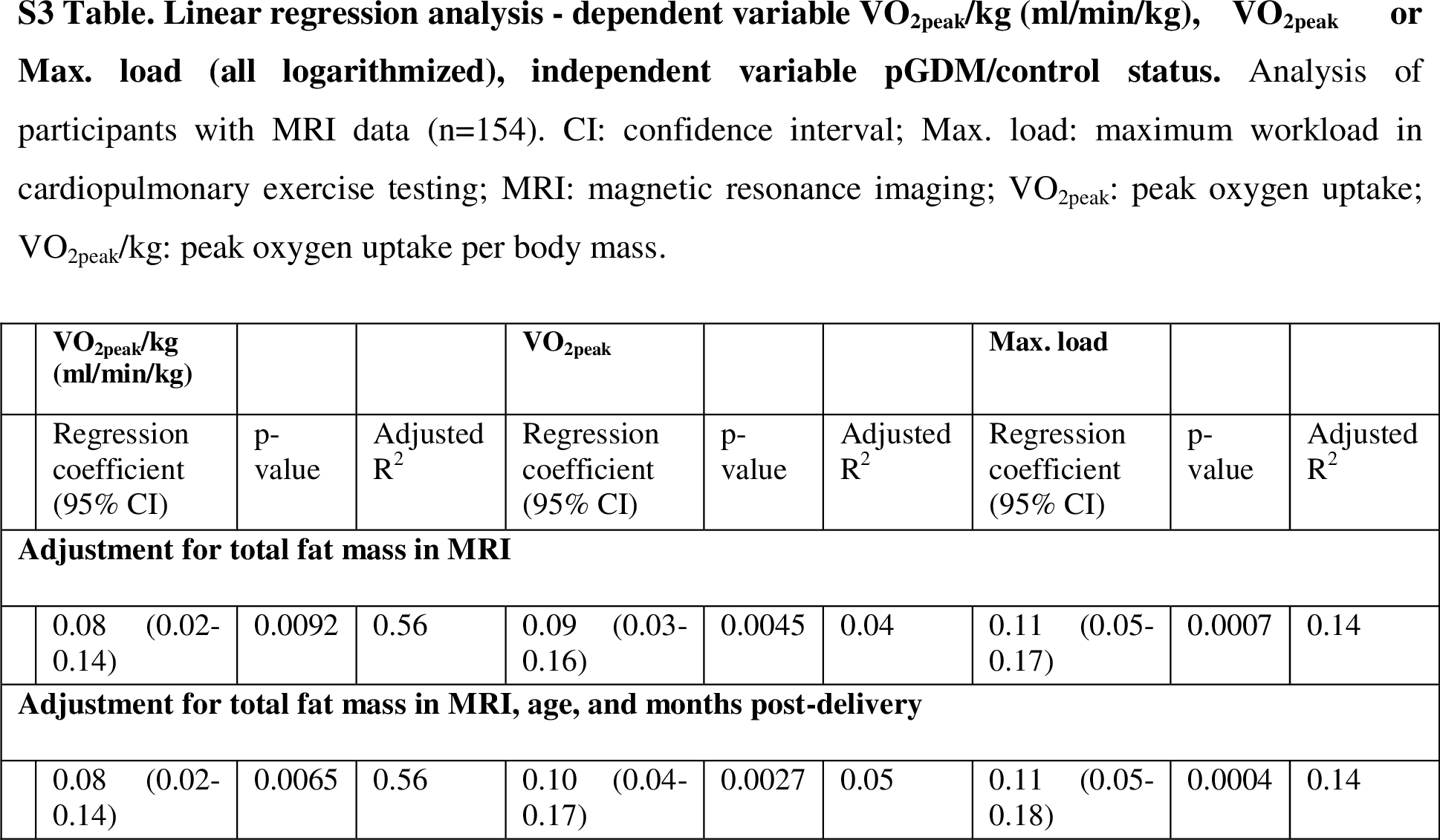

Supplement: S3 Table — Analysis of participants with MRI data (n = 154). CI: confidence interval; Max. load: maximum workload in cardiopulmonary exercise testing; MRI: magnetic resonance imaging; VO2peak: peak oxygen uptake; VO2peak/kg: peak oxygen uptake per body mass. (TIF) [file pone.0179128.s003.tif]

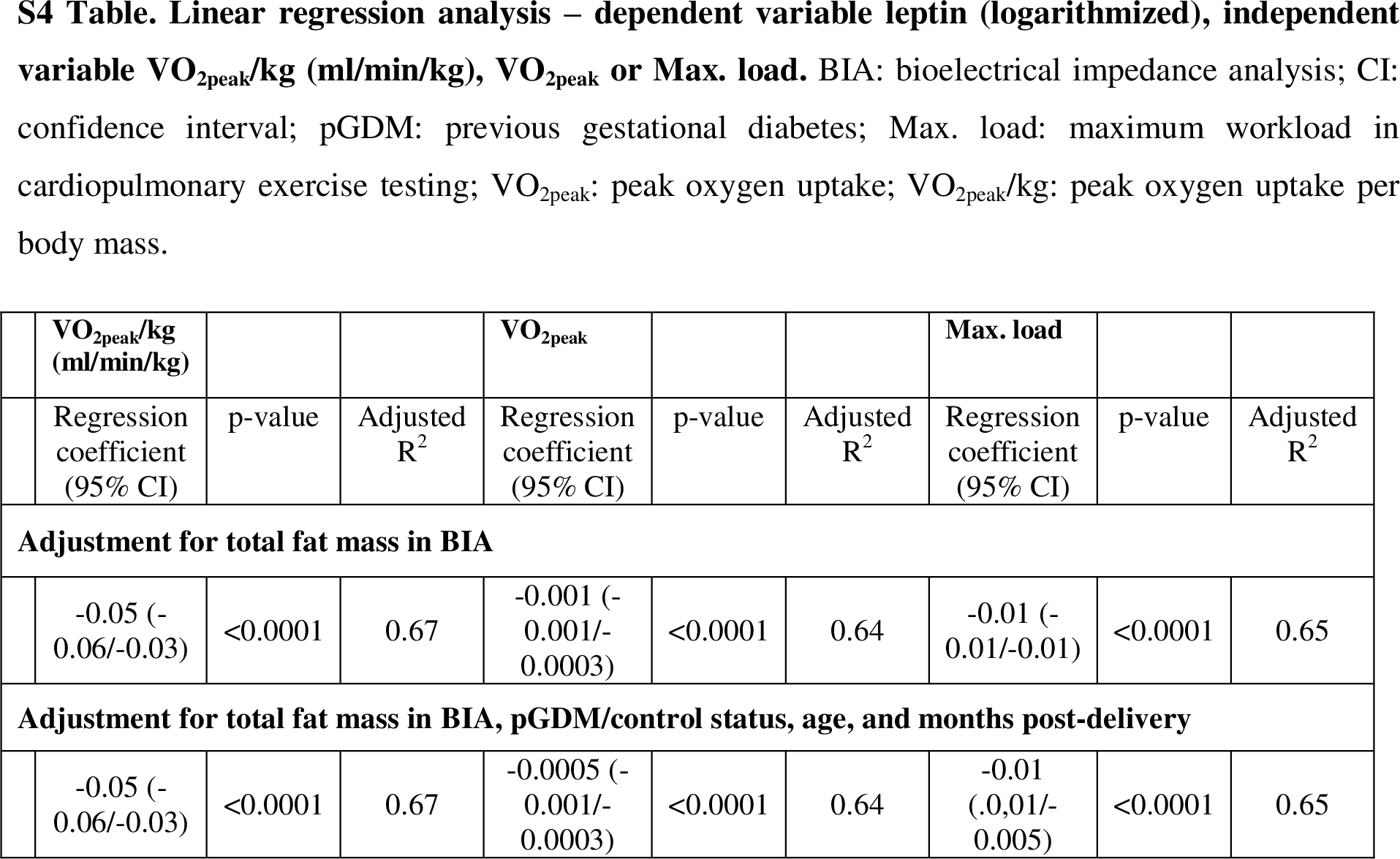

Supplement: S4 Table — BIA: bioelectrical impedance analysis; CI: confidence interval; pGDM: previous gestational diabetes; Max. load: maximum workload in cardiopulmonary exercise testing; VO2peak: peak oxygen uptake; VO2peak/kg: peak oxygen uptake per body mass. (TIF) [file pone.0179128.s004.tif]

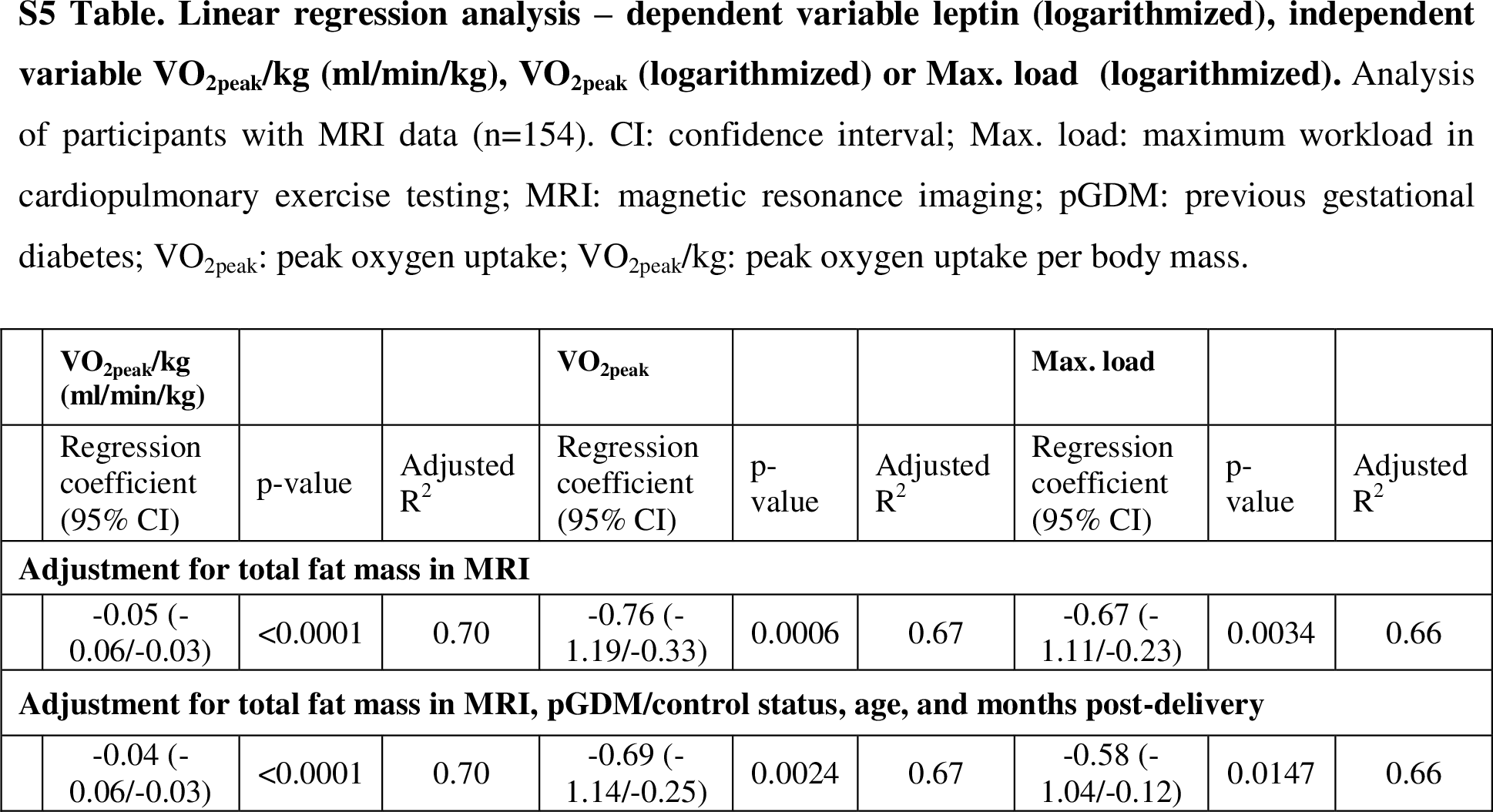

Supplement: S5 Table — Analysis of participants with MRI data (n = 154). CI: confidence interval; Max. load: maximum workload in cardiopulmonary exercise testing; MRI: magnetic resonance imaging; pGDM: previous gestational diabetes; VO2peak: peak oxygen uptake; VO2peak/kg: peak oxygen uptake per body mass. (TIF) [file pone.0179128.s005.tif]
